# Case Report: Auditory Neuropathy and Central Auditory Processing Deficits in a Neuro-Otological Case-Study of Infratentorial Superficial Siderosis

**DOI:** 10.3389/fneur.2020.610819

**Published:** 2021-01-14

**Authors:** Natallia Kharytaniuk, Peter Cowley, David J. Werring, Doris-Eva Bamiou

**Affiliations:** ^1^Ear Institute, University College London, London, United Kingdom; ^2^National Institute for Health Research, University College London Hospitals Biomedical Research Centre (Deafness and Hearing Problems Theme), London, United Kingdom; ^3^Department of Neuro-Otology, Royal National Throat, Nose and Ear Hospital, London, United Kingdom; ^4^Lysholm Department of Neuroradiology, National Hospital for Neurology and Neurosurgery, London, United Kingdom; ^5^Department of Brain Repair and Rehabilitation, Stroke Research Centre, Institute of Neurology, University College London, London, United Kingdom; ^6^Department of Neurology, National Hospital for Neurology and Neurosurgery, London, United Kingdom

**Keywords:** infratentorial superficial siderosis, central auditory deficits, auditory neuropathy, MRI, case report

## Abstract

Hearing and balance impairment are the most frequently reported features of infratentorial (classical) superficial siderosis (iSS). There are few comprehensive descriptions of audiovestibular function in iSS and therefore limited understanding of the affected segment(s) of the audiovestibular pathway. In addition, monitoring disease progression and response to treatment is challenging and currently mainly guided by subjective patient reports and magnetic resonance imaging. To the best of our knowledge, there have been no previous reports assessing central auditory function in iSS. We describe such findings in a patient with iSS in an attempt to precisely localize the site of the audiovestibular dysfunction, determine its severity and functional impact. We confirm the presence of (asymmetrical) auditory neuropathy and identify central auditory processing deficits, suggesting involvement of the central auditory pathway beyond the brainstem. We correlate the audiological and vestibular findings with self-report measures and the siderosis appearances on brain magnetic resonance images.

## Introduction

Infratentorial (classical) superficial siderosis (iSS) is a rare but increasingly recognized disabling neurological condition (1–3). It is characterized by haemosiderin deposition on the surfaces of the brain, cerebellum, brainstem and spinal cord due to chronic continuous or intermittent low volume and low pressure bleeding into the subarachnoid space (1). The most commonly identified cause of iSS is a dural defect, usually due to previous trauma or neurosurgery; the bleeding may originate from damaged capillaries at the dural breach margins (1, 2).

Clinically, iSS is characterized by a triad of hearing loss (most frequent symptom), imbalance (ataxia) and myelopathy. Hearing loss is usually described as high-frequency sensorineural, bilateral and often asymmetrical, ranging from mild-moderate to severe-profound (1, 3). It may resemble age-related hearing loss (ARHL) (1, 3, 4). The choice of audiological (and vestibular) tests is guided by the patient's signs and symptoms and the overall clinical presentation (4, 5). Reports of the auditory brainstem responses and stapedial reflexes findings are variable, with some also reporting cochlear involvement (3, 4). Balance dysfunction in iSS can be of both central (cerebellar) and peripheral vestibular origin (4, 5). Comprehensive systematic analysis of the audiovestibular function in iSS is lacking and it is difficult to ascertain the exact site of lesion (4). Central auditory function in iSS may be affected since haemosiderin is frequently deposited in the surfaces of key auditory processing areas including temporal cortices but there are no detailed studies (2, 4).

We report findings of central auditory dysfunction and bilateral (asymmetrical) auditory neuropathy in a patient with iSS and correlate these with the self-report measures and the brain magnetic resonance images (MRI). To our knowledge this is the first case-study to report central auditory processing testing in iSS in combination with structural neuroimaging and self-reports.

## Case Description

A 58 year-old male was referred with a radiologically confirmed diagnosis of iSS, likely from dural ectasia of the lumbosacral region. The patient reported a 4-year history of increasing difficulty hearing in noisy environments and a 3-year history of episodes of vertigo when standing or walking, a tendency to veer from the midline when walking and progressive imbalance when going uphill or on uneven surfaces. He had Marfan's syndrome, hypertension and was on warfarin for aortic valve replacement. There was no history of ear disease, noise exposure, head trauma, central nervous system tumors or surgery, and no family history of balance or hearing disorders.

Otoscopy was normal bilaterally. There was left-sided primary position esotropia (present since childhood) and reduced upgaze and medial gaze eye movements, no nystagmus, normal smooth pursuit, saccades and finger-nose test. The patient had a broad-based gait, mild heel-shin dysmetria and was unable to perform tandem walk, Romberg's or Unterberger's-tests.

### Audiological Testing

**Figure 1:**
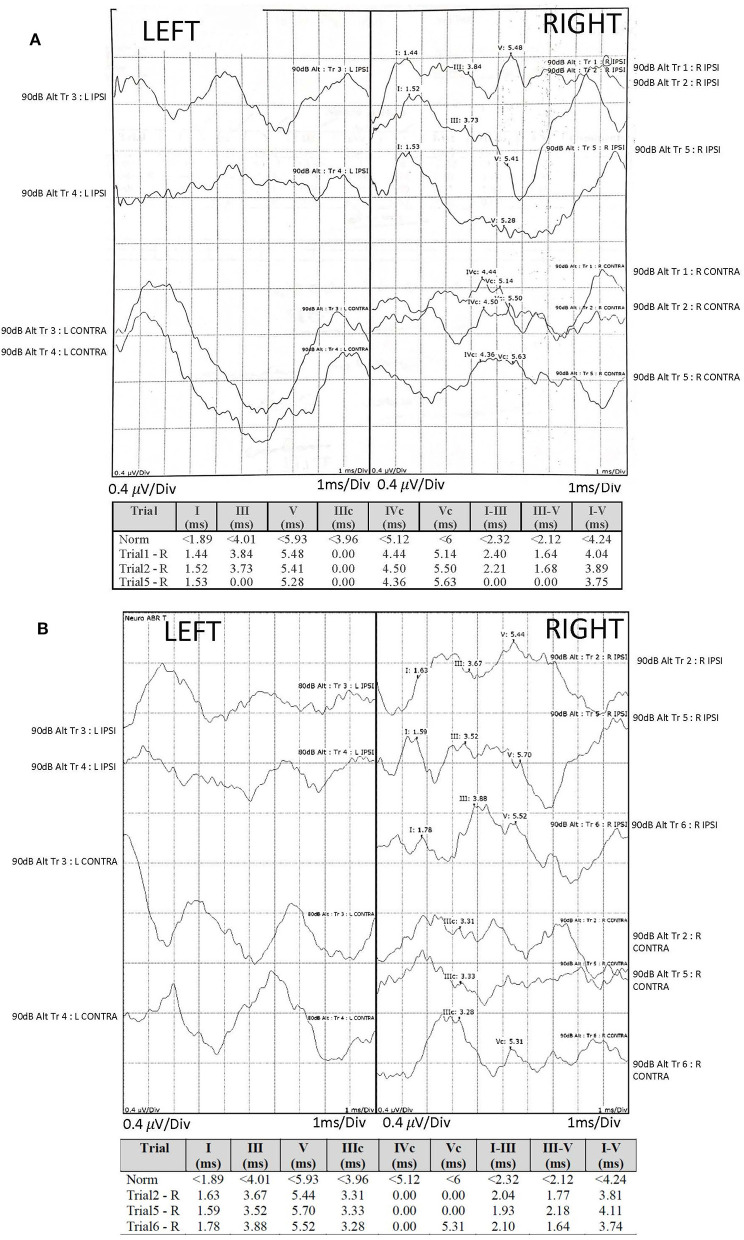
Audiological assessment: auditory brainstem responses (ABR) were recorded using TDH-39 headphones and monaurally presented alternating polarity click stimuli of 100 μs duration, 11.33 Hz repetition rate and intensity of 90 dB nHL (normalized hearing level). The electrodes were mounted on center forehead (common); A1 left mastoid (active), A2 right mastoid (active) and high center forehead (reference). The responses were compared with our institutional normative values (provided) and demonstrated reproducible right waves I-V of degraded morphology, yet normal amplitude and latency and absent left responses at baseline **(A)** and at a 6-month interval **(B)**, with additional findings of poorly reproducible right waves I-V and poor wave I morphology at a 6-month interval **(B)**. Due to retrospective nature of the case report, it was impossible to separate the recordings into condensation and rarefaction buffers and to comment on the cochlear microphonic potentials.

Pure-tone audiometry, speech recognition tests, transient evoked otoacoustic emissions, and auditory brainstem responses were performed to assess auditory function up to the brainstem (Figure 1 and Table 1). Middle ear involvement was ruled out with normal tympanometry. Pure-tone audiometry showed mild-to-moderate high-frequency sensorineural hearing loss attributable to age-related changes (6). Transient evoked otoacoustic emissions were reduced at 4 kHz bilaterally consistent with pure-tone audiometry findings. Speech recognition thresholds were elevated compared to pure-tone thresholds, indicating bilateral auditory neuropathy.

Table 1Results of auditory assessment and self-report audiovestibular measures.

**Table:** 

**A. Peripheral auditory tests**									
**Pure-tone audiometry**	**Frequency (kHz)**								
	**0.25**	**0.5**	**1**	**2**	**4**	**6**	**8**	**3FA (%)**	**4FA (%)**
**Baseline:**									
**Right AC (dB)**	15	15	10	15	25	55	75	13.3 (50)	16.25 (50)
**Left AC (dB)**	20	15	25 BC^*^	20	35 BC^*^	55	70	20 (80)	23.75 (75)
**6-month interval:**									
**Right AC (dB)**	25	25	15	20	30	-	60	20 (85)	22.5 (75)
**Left AC (dB)**	20	20^*^	25	25^*^	30^*^	40	70	23.3 (90)	25 (75)

**Table:** 

**Transient evoked otoacoustic emissions**	**Frequency (kHz)**				
	**1.0**	**1.4**	**2.0**	**2.8**	**4.0**
**Signal-to-noise ratio: Right (dB)**	7.0	13.8	10.4	11.2	5.4
**Signal-to-noise ratio: Left (dB)**	16.9	16.6	21.7	6.0	1.7

**Table:** 

**Speech in quiet audiometry testing**		
Speech recognition threshold (dB HL)	55 (right)	75 (left)
Most comfortable level (dB HL)	35 (right)	45 (left)

**Table:** 

**B. Central auditory processing tests (baseline)**			
**Test**	**Right**	**Left**	**Normal limits**
**Dichotic digits test (% correct)**	30%	30%	≥90% (normal hearing subjects) ≥80% (cochlear hearing loss)
**Frequency pattern test (% correct)**	40%	53%	≥80%

**Table:** 

**Listening in spatialized noise-sentences test**				
**Measure**	**Age average score (dB)**	**Cut-off score (dB)**	**Patient's score (dB)**	**Variants from average (SD)**
Low-cue SRT	−0.2	1.7	7.5	−8.0
High-cue SRT	−13.0	−8.9	8.5	−10.3
Talker advantage	9.1	4.7	2.9	−2.9
Spatial advantage	12.0	8.7	0.1	−7.2
Total advantage	13.3	9.4	−1.0	−7.3
**Listening in spatialized noise-sentences test PGA results**				
High-cue SRT	−13.0		8.5	21

**Table:** 

**C. Self-report questionnaires**	**Patient's score**	**Normal values**
***Modified Amsterdam inventory for auditory disability and handicap (mAIADH)***		
Speech intelligibility in noise	0	Maximum best 15
Speech intelligibility in quiet	6	Maximum best 15
Auditory Localization	2	Maximum best 15
Detection of sounds	12	Maximum best 15
Distinction of sounds	9	Maximum best 24
Total score	29	Maximum best 84
***Speech spatial qualities of hearing scale questionnaire (SSQ)***		
Subscales scores: Speech	0.92	14 questions
Spatial	1.23	17 questions
Quality	1.6	18 questions
Total score	3.75	49 questions in total
***Dizziness handicap inventory (DHI)***		
Subscales scores: Physical	16	>20 significant impact on lifestyle
Functional	24	16–34 (mild)
Emotional	32	36–52 (moderate handicap)
Total composite score	72	54+ (severe)
**Situational vertigo questionnaire (SVQ)**	0.39	Visual vertigo if score > 1

**(A)** peripheral auditory tests: pure-tone audiometric thresholds at baseline and at 6 months demonstrating near-normal peripheral auditory function, with mild deterioration in thresholds at 6 months; 3-frequency/4-frequency averages were compared with normative values (percentiles) and were age-/ear-/sex-matched (51–60, right/left, male), not specific to occupation and no noise-exposure or air-bone gap > 0 (6). Cochlear function was unimpaired as indicated by the presence of transient evoked otoacoustic emissions at frequencies 1.0–2.8 kHz and reduced at 4 kHz bilaterally, speech recognition thresholds were worse than expected for pure-tone averages; **(B)** central auditory processing tests: dichotic digits and frequency pattern recognition tests scores were markedly below the normal values for both tests. Calculated Listening in Spatialized Noise-Sentences test scores were below two standard deviations of the norm and a signal-to-noise ratio of 21.5 dB was required to understand speech almost as well as for people with normal hearing. The loss of speech understanding in noise was graded as severe; **(C)** self-report auditory (mAIADH, SSQ) and vestibular (DHI, SVQ) questionnaires demonstrating reduced scores for all, except for situational vertigo questionnaire. AC, air conduction; BC, bone conduction; dB, decibel; kHz, kilohertz; SD, standard deviation; SRT, speech reception thresholds; PGA, prescribed gain amplifier; 3FA, three-frequency average (0.5/1.0/2.0 kHz); 4FA, four-frequency average (0.5/1.0/2.0/4.0 kHz);

* *masked*.

Auditory brainstem responses (compared against our normative values) demonstrated reproducible right waves I-V of degraded morphology, yet normal amplitude and latency and absent left responses. Absent left responses persisted on 6-month interval testing, with additional findings of poorly reproducible right waves I-V and poor wave I morphology, suggesting progressive right auditory nerve involvement.

Measures of central auditory function included dichotic digits test, (monaurally presented) frequency pattern test and Listening in Spatialized Noise-Sentences test. Dichotic digits test score was calculated as percent correct of sets of four digits presented simultaneously (two digits on each side). Frequency pattern test score was calculated as percent correct of the recognized sequences of three tone bursts of high and low frequency. The stimuli for both tests were presented at 50 dB SL (sensation-level) above the threshold level. Listening in Spatialized Noise-Sentences test scores were calculated by the software based on correct recognition of stimuli (sentences) presented simultaneously with competing sentences presented at 55 dB SPL (sound-pressure-level) of the same and different speaker-voice and at azimuth and 90 degrees to the stimuli sentences at a stimulus presentation level determined by the software. The scores were adjusted for mild peripheral hearing loss and were markedly low (Table 1).

### Vestibular Testing

**Figure 2:**
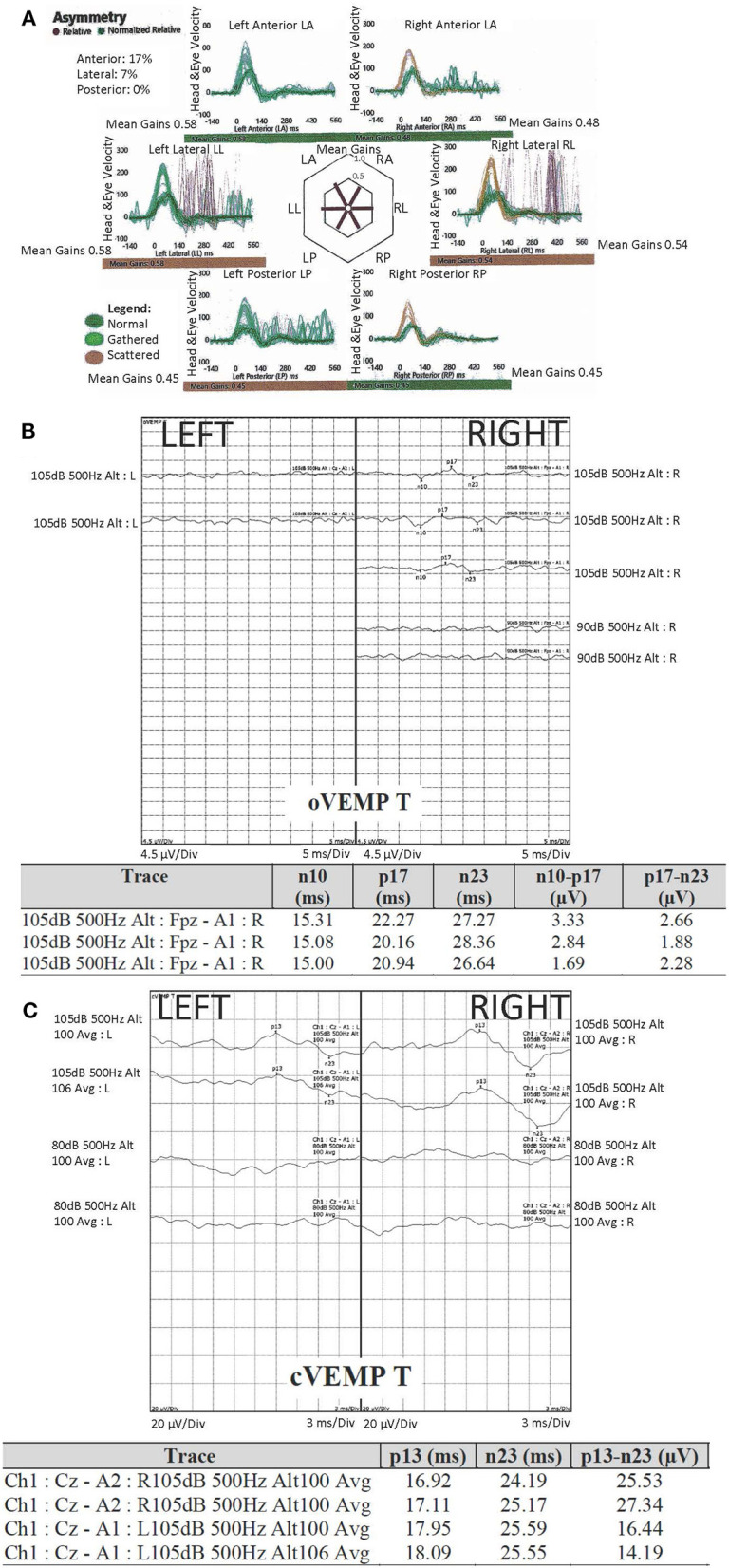
Peripheral vestibular assessment: **(A)** video head impulse Test (vHIT), demonstrated reduced gain in all six canals, more marked in the right anterior (asymmetry 17%) and both posterior canals; **(B)** ocular vestibular evoked myogenic potentials (oVEMP) were not detected on the left; **(C)** cervical vestibular evoked myogenic potentials (cVEMP) were compared to our normative values and were within normal limits. Tone burst stimuli of 500 Hz was used for both oVEMP and cVEMP, of alternating polarity (specific to the unit's equipment) and 2:1:2 cycle. The scale was 5 ms per division (oVEMP) and 3 ms per division (cVEMP). For oVEMP, the electrodes were placed on forehead (common), A1 centrally below the eye; REF (reference) electrode on the cheek 1 cm below (but not touching) A1. For cVEMP, the electrodes were placed on forehead (common), sternoclavicular joint (inverting/negative), sternocleidomastoid muscle belly (non-inverting/positive).

Peripheral vestibular tests included cervical and ocular vestibular evoked myogenic potentials and video head impulse test, whereas videonystagmography was performed to distinguish between the peripheral and central vestibular involvement and to assess severity of the dysfunction (Figure 2). Video head impulse testing identified reduced mean vestibulo-ocular reflex gains for all six canals (Figure 2A). Left ocular vestibular evoked myogenic potentials were not detected (Figure 2B). Cervical vestibular evoked myogenic potentials were within normal limits (Figure 2C). Videonystagmography showed impaired gaze holding to the left and minimally impaired smooth pursuit at 0.4 Hz only, indicating mild central vestibular involvement (7–10). Vestibular test values were compared against our normative values where available.

### Neuro-Psychological Assessment and Self-Report Measures

The patient completed validated hearing- and balance-specific questionnaires. Modified Amsterdam Inventory for Auditory Disability and Handicap (28 items) and Speech, Spatial and Qualities of Hearing Scale (49 items), were used to assess hearing difficulties attributable to everyday situations in five domains (sound recognition, detection and localization, and speech intelligibility in quiet and in noise) (11, 12), and in the domains of speech, spatial and qualities of hearing, respectively (13). Dizziness Handicap Inventory (25 items) was used to assess functional, emotional and physical impact of imbalance and vertigo on the patient's quality of life (14), whereas Situational Vertigo Questionnaire (19 items) assessed severity of vestibular symptoms in visually disorienting situations (15).

The self-report measures scores were consistent with severe disability, except for normal Situational Vertigo Questionnaire score (Table 1). Neuro-psychological assessment was performed and the scores were within low average range except for visual recognition and phonemic fluency impairment and minimal attentional and executive inefficiency.

### Imaging

**Figure 3:**
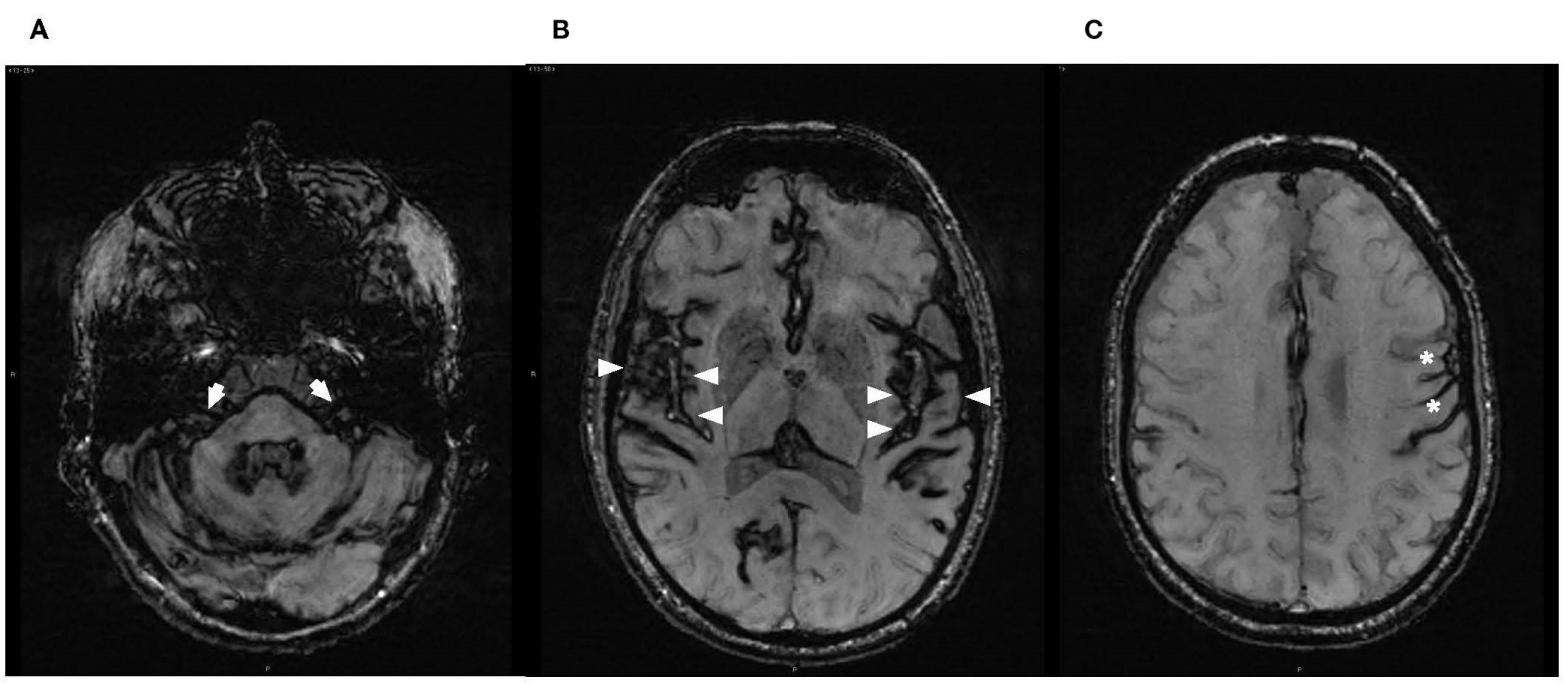
Axial susceptibility-weighted magnetic resonance images (SW-MRI) at the level of **(A)** brainstem/internal auditory canals, demonstrating haemosiderin deposition over the pons and vestibulocochlear nerves bilaterally with slightly thicker hypointense rim of hemosiderin on the left (arrows); **(B)** superior temporal gyri, demonstrating supratentorial haemosiderin deposition along the surfaces of the temporal lobes, involving insular areas and particularly Sylvian fissures and Heschl's gyri (arrowheads); and **(C)** asymmetry in the supratentorial appearance of haemosiderin deposition on the left (asterisks).

The brain and spine MRI were performed with MAGNETOM SKYRA 3T (Siemens, UK). Susceptibility weighted imaging sequences (SWI-MRI, 3D-T2* GRE, 1.5 mm) identified appearances of haemosiderin deposits consistent with the radiological diagnostic criteria for iSS (2). It demonstrated hypointense regions along the cerebellar folia and superior vermis, midbrain, pons and both vestibulocochlear nerves (more marked on the left), medulla and cranio-cervical junction. There was additional involvement of supratentorial structures, with severe and widespread siderosis affecting the surfaces of temporal lobes, including Sylvian fissures and insular areas, frontal and occipital lobes, but sparing the vertex (Figure 3).

### Management

Following the Siderosis Multidisciplinary Team meeting, the patient was commenced on Deferiprone, as there was no clear neurosurgical target for dural repair. The patient was aware of the risks associated with Deferiprone and the need for regular blood monitoring for neutropenia. He was referred for hearing therapy as a rehabilitative measure in view of central auditory deficits with near-normal peripheral auditory function, and for neuro-vestibular physiotherapy to address mixed (central/peripheral) vestibular deficits. There were no adverse events, such as neutropenic sepsis. The treatment was suspended in view of COVID19 risks. He has a regular follow-up with Neurology, Neuro-otology and Hematology teams.

## Discussion

A significant finding in this case report is the presence of central auditory processing deficits. It is important not only for diagnosis and understanding of the clinical spectrum of iSS but also for the potential approach to treatment.

Central auditory involvement was indicated by the bilaterally reduced scores for frequency pattern and dichotic digits tests (Table 1). These could not be attributed to auditory neuropathy alone as they previously showed correlation with cortical lesions (16, 17) and are known to be robust against mild-to-moderate cochlear hearing loss (16, 18). Cerebral involvement was further indicated by reduced scores for Listening in Spatialized Noise-Sentences test, particularly in low and high cues and spatial advantage domains which could not be attributed to auditory neuropathy alone (Table 1) (19, 20). The findings of difficulty integrating spatial auditory information and impairment of temporal processing were previously reported in individuals with auditory neuropathy in Friedreich's Ataxia (FRDA) and Charcot Marie Tooth (CMT) disease (Type 1A) (20). Yet, in our own FRDA cohort (21), we found abnormal spatial advantage on Listening in Spatialized Noise-Sentences test even in cases with normal auditory brainstem responses and conversely a case with abnormal responses yet normal spatial advantage. This is possibly consistent with the reports of cerebral cortical atrophy in the auditory brain areas in FRDA (22). Thus, the abnormal Listening in Spatialized Noise-Sentences test findings are likely to be congruent with central auditory involvement, and in particular the antero-lateral aspect of Heschl's gyrus (23), rather than the fronto-temporal and fronto-parietal cortical network as our patient's memory functions were intact.

Our case highlights the shortcomings of pure-tone audiometry as a single tool for hearing assessment. Marked hearing difficulties reported by our patient were not consistent with mildly elevated (likely age-related) thresholds (24, 25) as further testing including speech recognition thresholds identified asymmetrical auditory neuropathy and central auditory processing deficits (20, 26). Comprehensive test battery should be performed for patients with auditory symptoms that are more marked than their peripheral auditory test results. This is in line with the current audiological guidelines (27, 28) and the recommended neurological work-up for patients with other neurodegenerative disorders such as FRDA and CMT (20, 29).

Our findings also build on the previous reports of mixed vestibular involvement (8–10). ISS should be considered as a differential diagnosis in patients with chronic combined vestibulopathy (30). It is plausible that the vestibular hypofunction may inform an iSS-specific pattern for vestibular involvement, with preferentially lower gains for posterior semi-circular canals on video head impulse testing, as seen in our patient (9, 31, 32). Although the interpretation of ocular vestibular evoked myogenic potentials should be done with caution—due to the patient's restricted upward gaze—the responses were not detected on the left which may be consistent with the involvement of the left superior vestibular nerve. This finding may be further commensurate with the absent left ABR (33).

In a case-series of five patients with superficial siderosis, the superior vestibular nerve and utricle involvement were identified in all five cases (34). Three patients had a protracted course and showed additional involvement of the inferior vestibular nerve and saccule. It was hypothesized that the superior vestibular nerve involvement may occur in earlier stages of iSS, with gradual disease progression leading to the inferior vestibular nerve involvement (5).

Although the identified vestibulopathy may be associated with vestibular nerve involvement and sparing of saccule and utricle (35, 36), vestibulocochlear end-organ damage was previously reported in iSS (5, 34, 37). In the same case series, the authors suggested that the vestibular loss in superficial siderosis was likely to be due to impaired blood flow to the vestibulocochlear apparatus rather than damage to the vestibular nerves. This cannot be supported by the presence of otoacoustic emissions in our patient, as outer hair cells are very susceptible to hypoxia (38–41).

Involvement of vestibulocochlear apparatus in iSS cannot be supported by the single report of temporal bone histology in a patient with superficial siderosis (42). The authors described atrophy of the strial ganglia, absence of hair cells only in the cochlear basal turn bilaterally, iron deposits in stria vascularis and spiral ligament and the subepithelial layers of macula, and a marked atrophy of the vestibulocochlear nerve (42). It is, difficult to conclude that those histological findings could be solely siderosis-related, but perhaps due to several pathological processes in the inner ear and along the auditory pathway, likely from the reported noise exposure and identified otosclerosis (42). Stria vascularis and spiral ligament are highly vascularized structures, and the presence of ferritin in stria vascularis (with a proposed possible function of stria vascularis for iron storage) was reported (43, 44).

Cochlear aqueduct patency was proposed as a mechanism for hearing loss in iSS (37) which would imply damage to the inner ear apparatus through altered biochemical composition of perilymph or direct effect of haemosiderin and iron by-products. Damage to the inner ear sensory and neural apparatus was not evident in a study of 12 temporal bones of patients who died from subarachnoid hemorrhage (45).

Equally, it is important to consider hearing loss in the setting of Marfan's syndrome which has been described, albeit infrequently, as predominantly conductive, associated with otitis media, Eustachian tube dysfunction and cranio-facial abnormalities (46). Sensorineural hearing loss in this group may be due to hypertension, thus resulting in cochlear vascular damage and sensory hearing loss (46–48). This is in contrast to the findings in our patient as presence of otoacoustic emissions indicated near-normal cochlear function and normal middle ear conduction. It is possible that the vestibulocochlear apparatus damage, described in other studies, might be due to age-related changes, presence of other risk factors for hearing and balance impairment, and possibly the anterograde progression of auditory and vestibular dysfunction.

The MRI appearance of hypointense regions along the course of both vestibulocochlear nerves (slightly more marked on the left) was consistent with the clinical findings of asymmetrical auditory neuropathy and concurrent vestibulopathy (Figure 3). Severe auditory processing deficits in our patient may be commensurate with the cerebral abnormalities involving the auditory cortex as evidenced by the MRI appearance of hypointensities over the cortical surfaces involving Sylvian fissures, superior temporal gyri and the insulae (Figure 3).

Scores of dedicated self-report measures were consistent with the identified auditory and vestibular deficits (Table 1). The total score for modified Amsterdam Inventory for Auditory Disability and Handicap questionnaire was below the reported scores for patients with mildly elevated mean audiometric thresholds, for those undergoing tympanoplasty (11, 49) and for patients with auditory processing disorder (50). The overall Speech Spatial and Qualities of Hearing Scale score was similar to the scores reported for hearing-impaired individuals (51, 52). These two self-report measures were previously shown to correlate with auditory processing deficits in adults with normal hearing thresholds, although not in a neurological population (50). The total Dizziness Handicap Inventory score of 72 (severe) (14) was comparable to scores for individuals with benign paroxysmal positional vertigo (BPPV) yet worse than in central, bilateral peripheral or mixed vestibular dysfunction and traumatic brain injury (7, 53–55). In contrast, the Situational Vertigo Questionnaire score of 0.39 was similar to the scores for normal individuals (56), most probably indicating little-to-no impact of visual stimuli on our patient's vestibular symptoms.

Management of auditory processing disorder and ataxia in complex neurological patients requires individualized approach and is deficits- and needs-specific. Listening strategies, use of assistive listening devices, auditory training, hearing aids and cochlear implants (where fitting the criteria) should be considered. Reported outcomes of cochlear implantation in iSS patients are variable (57, 58) but may improve with meticulous auditory evaluation and precise site-of-lesion identification. Dedicated neuro-vestibular physiotherapy previously demonstrated benefits (59) and should be prescribed for patients with complex vestibular impairment to address their functional deficits based on the lesion location.

There are several learning points in our case-study. We identified central auditory processing deficits in iSS, which correlate with the MRI findings and scores for self-report measures. Our case-study provides clear evidence of auditory and (concurrently) vestibular neuropathy, without confounding risk factors for end-organ involvement. It is possible that the anterograde progression of audiovestibular dysfunction may result in end-organ involvement. We highlight the need for comprehensive audiovestibular assessments in patients with neurodegenerative conditions to identify the site of lesion and provide patient- and deficit-specific management strategies.

The limitations of this case-study are in its level/strength of evidence. Further dedicated prospective studies are needed to investigate whether the findings are patient-specific or are characteristic for iSS. Although there was stark asymmetry between right and left ocular vestibular evoked myogenic potentials, the results should be interpreted with caution in view of patient's restricted upgaze and previous reports of absent potentials in normal individuals (60).

Patient's perspective: the patient's referral to our tertiary center, dedicated investigations and multidisciplinary input helped his understanding of the condition and of his symptoms. While he perceived little benefit from physiotherapy, with further gradual deterioration in balance, he reported benefit from management of his hearing deficits.

## Conclusion

There have been no previously documented central auditory function assessments for patients with iSS and correlation with the scores of self-report measures and MRI findings. Our case-study provides clear evidence of auditory and (concurrently) vestibular neuropathy, without confounding risk factors for end-organ involvement. It is possible that the end-organ involvement may be the result of anterograde progression of audiovestibular dysfunction. The importance of comprehensive audiovestibular assessments to identify the site of lesion in patients with neurodegenerative conditions is highlighted. Due to progressive, and possibly irreversible, nature and significant morbidity, it is necessary to determine the features that differentiate iSS-related and age-related hearing loss for timely diagnosis and to provide needs- and deficit-specific management strategies, with further efforts to halt disease progression by means of prompt surgical repair (61, 62), or by using iron chelating agents (1, 63, 64) and ultimately, may inform research on novel therapeutic agents. Dedicated and longitudinal studies that correlate the audiovestibular assessments, patients' symptoms and the degree of functional impairment with the respective imaging would help establish MRI usefulness in functional assessment of patients with iSS. It is possible that MRI may lack sensitivity in determining subtle changes associated with disease progression or treatment response. It is plausible that serial audiovestibular testing, alongside self-report measures, may be more useful in identifying such changes and appropriate in the setting of neuro-otology clinic without invasive testing or MR imaging.

## Data Availability Statement

The original contributions generated in the study are included in the article/supplementary materials, further inquiries can be directed to the corresponding author.

## Ethics Statement

Ethical review and approval was not required for the study on human participants in accordance with the local legislation and institutional requirements. The patients/participants provided their written informed consent to participate in this study. Written informed consent was obtained from the individual(s) for the publication of any potentially identifiable images or data included in this article. Formal written consent was sought from the patient prior to the write up and submission of the manuscript, including consent for the publication of any potentially identifiable images or data included in this article.

## Author Contributions

NK wrote the manuscript. D-EB contributed to content and manuscript writing. DJW and PC reviewed the manuscript and provided comments. All authors reviewed the final version of the manuscript.

## Conflict of Interest

The authors declare that the research was conducted in the absence of any commercial or financial relationships that could be construed as a potential conflict of interest.
